# Baroreflex Curve Fitting Using a WYSIWYG Boltzmann Sigmoidal Equation

**DOI:** 10.3389/fnins.2021.697582

**Published:** 2021-09-27

**Authors:** Karsten Heusser, Ramona Heusser, Jens Jordan, Vasile Urechie, André Diedrich, Jens Tank

**Affiliations:** ^1^Institute of Aerospace Medicine, German Aerospace Center, Cologne, Germany; ^2^Immanuel Kant High School, Wilthen, Germany; ^3^University of Cologne, Cologne, Germany; ^4^Division of Clinical Pharmacology, Department of Medicine, Autonomic Dysfunction Center, Vanderbilt University Medical Center, Nashville, TN, United States

**Keywords:** baroreflex curve, baroreflex gain, baroreflex sensitivity, RR interval, muscle sympathetic nerve activity, sigmoidal curve fitting, Boltzmann sigmoidal equation

## Abstract

Arterial baroreflex assessment using vasoactive substances enables investigators to collect data pairs over a wide range of blood pressures and reflex reactions. These data pairs relate intervals between heartbeats or sympathetic neural activity to blood pressure values. In an X-Y plot the data points scatter around a sigmoidal curve. After fitting the parameters of a sigmoidal function to the data, the graph’s characteristics represent a rather comprehensive quantitative reflex description. Variants of the 4-parameter Boltzmann sigmoidal equation are widely used for curve fitting. Unfortunately, their ‘slope parameters’ do not correspond to the graph’s actual slope which complicates the analysis and bears the risk of misreporting. We propose a modified Boltzmann sigmoidal function with preserved goodness of fit whose parameters are one-to-one equivalent to the sigmoidal curve’s characteristics.

## Introduction

Baroreflexes play an important role in the regulation of the circulatory system. As negative feedback systems they stabilize arterial pressure around the so-called operating pressure. This feature is also known as blood pressure buffering to prevent large deviations from its setpoint. Often cardiovascular diseases are associated with impaired baroreflexes. Therefore, baroreflex quantification may be useful to assess the current state, progression, and therapeutic improvements of cardiovascular diseases. Moreover, precise baroreflex measurements can help unravel complex physiologic or pharmacologic mechanisms (Heusser et al., 2016).

Although there are numerous methods to evaluate baroreflex function they all share the same basic principle of relating the reflex response (output) to the stimulus intensity (input). Typically, systolic arterial pressure is taken as input and heartbeat interval (RR interval, RRI) as output. The quantitative relationship between these parameters is used to characterize the so-called cardiac or vagal or cardiovagal baroreflex. Baroreflex gain or sensitivity is the most reported index. Values range from virtually zero in patients with complete baroreflex failure (Heusser et al., 2005) up to 40 ms/mmHg in trained athletes (Baumert et al., 2006). In cardiovascular laboratories, investigators may be interested in baroreflex mechanisms over a wide range of blood pressures that does not only include the linear part of the stimulus–response relationship but also saturation and threshold portions (Parati et al., 2000). Such data can be obtained by injection or infusion of the vasoactive substances sodium nitroprusside (vasodilator) and phenylephrine (vasoconstrictor). In an X-Y plot, data pairs relating RRI or sympathetic neural activity to blood pressure readings scatter around sigmoidal curves.

**FIGURE 1:**
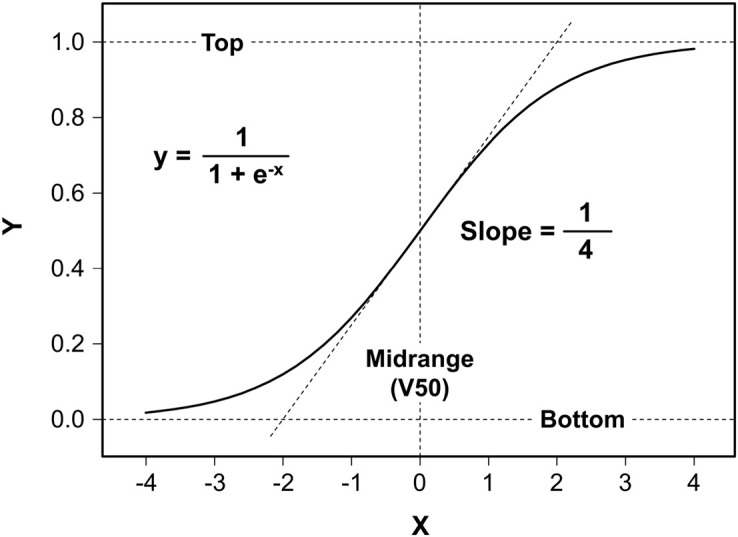
Logistic function. The logistic function (without any parameters) represents the prototype of widely used functions for sigmoidal curve fitting to appropriate classes of two-dimensional experimental data. The graph has four characteristic values: Bottom = 0.0, Top = 1.0, maximum Slope = 0.25 at Midrange = 0.0, which is the abscissa of the curve’s central inflection point.

Logistic functions (Verhulst, 1838) are widely used for sigmoidal curve fitting, whose prototype was invented to describe population growth with saturation (Figure 1). This function has four characteristic values: Bottom = 0.0, Top = 1.0, and maximum Slope = 0.25 at Midrange = 0.0.

**FIGURE 2:**
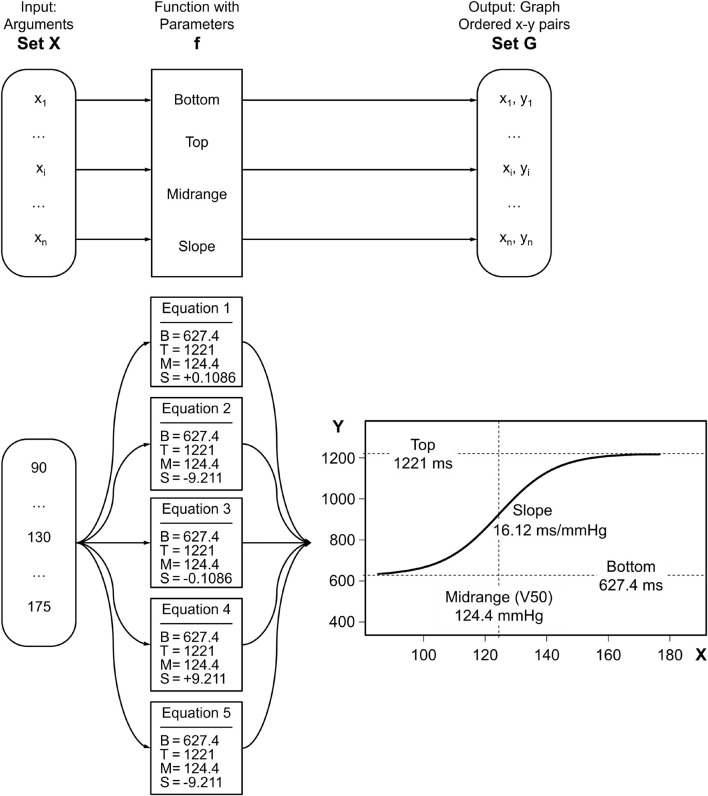
Discrepancy between the equations’ Slope parameters and their graphs’ slopes. The five function boxes in the middle column of the figure implement the versions of the 4-parameter logistic equations in Table 1. The parameters [B]ottom, [T]op, and [M]idrange are identically set for all functions while [S]lope settings differ. However, the functions’ *graphs* are identical. In other words, the curves representing them are exactly superposed (bottom right). Their identical Slopes at the inflection point at Midrange are 16.12. Hence, there is a discrepancy between [S]lope *parameter* settings in the functions (middle column) and the functions’ *graphs* (on the right).

Most real data that follow a sigmoidal X-Y relationship have other characteristics than the prototypic logistic function. For example, as can be seen from the curve in Figure 2, RR intervals show asymptotic behavior against a lower and upper limit (Bottom and Top) of the baroreflex response which are different from 0.0 and 1.0 of Verhulst’s logistic function. Likewise, the abscissa value of the central inflection point (Midrange) and the slope of the graph at that point (Slope) vary from Verhulst’s values, 0.0 and 0.25, respectively. In 1972, Kent et al. proposed a generalized 4-parameter logistic function, often referred to as Boltzmann sigmoidal equation, to model the baroreflex relationship between systemic arterial and carotid sinus pressure (see Equation 1 in Table 1). Kent et al. used A1..A4 as parameter names. In the following, we will use the more informative terms [B]ottom, [T]op, [R]ange (= T–B), [S]lope, and [M]idrange (or V50). Numerous investigators have applied the equation in its original form or variants thereof for sigmoidal curve fitting to their two-dimensional data (Table 2). The usefulness of the method has been confirmed for decades. It should be particularly emphasized that all 4-parameter equation variants yield absolutely identical output after ideal fitting of the 4 parameters. In other words, after successful fitting of the equations’ parameters to the same data their graphs would perfectly overlap. This statement applies to the traditional equations as well as our modified Equation 8 which will be presented in the Methods section.

**TABLE 1:** Versions of 4- and 5-parameter Boltzmann sigmoidal equations.

Specifics	No explicit range parameter	Explicit range parameter
Equation 1: Exponent S(x–M)	$y=f\left(x\right)=B+\frac{T-B}{1+e^{S\left(x-M\right)}}$	$y=f(x)=B+\frac{R}{1+e^{S\left(x-M\right)}}$
Equation 2: Exponent (x–M)/S	$y=f\left(x\right)=B+\frac{T-B}{1+e^{\frac{\left(x-M\right)}{S}}}$	$y=f\left(x\right)=B+\frac{R}{1+e^{\frac{\left(x-M\right)}{S}}}$
Equation 3: Exponent S(M–x) = –S (x–M)	$y=f\left(x\right)=B+\frac{T-B}{1+e^{S\left(M-x\right)}}$	$y=f\left(x\right)=B+\frac{R}{1+e^{S\left(M-x\right)}}$
Equation 4: Exponent (M–x)/S	$y=f\left(x\right)=B+\frac{T-B}{1+e^{\frac{\left(M-x\right)}{S}}}$	$y=f\left(x\right)=B+\frac{R}{1+e^{\frac{\left(M-x\right)}{S}}}$
Equation 5: Absolute term T instead of B	$y=f\left(x\right)=T+\frac{B-T}{1+e^{\frac{\left(M-x\right)}{S}}}$	$y=f\left(x\right)=T+\frac{-R}{1+e^{\frac{\left(M-x\right)}{S}}}$
Equation 6: [A]symmetry parameter added		$y=f\left(x\right)=B+\frac{R}{{\left(1+e^{S\left(x-M\right)}\right)}^{A}}$
Equation 7: Sigmaplot’s asymmetric function		$y=f\left(x\right)=B+\frac{R}{{\left(1+e^{S\left(M-x\right)}\right)}^{A}}$

**TABLE 2:** Selected references referring to variants of the 4-parameter Boltzmann sigmoidal equation.

Equation 1	Kent et al., 1972; Dorward et al., 1985; Verberne et al., 1987; Rocchiccioli et al., 1989; Saad et al., 1989; Shade et al., 1990; Itoh and van den Buuse, 1991; Kawada et al., 1992; Martel et al., 1994; Veelken et al., 1994; Bartholomeusz and Widdop, 1995; Schenberg et al., 1995; Sagawa et al., 1997; He et al., 1999; Sampaio et al., 1999; Ma et al., 2002; Bealer, 2003; Miki et al., 2003; Cheng et al., 2004; Nagura et al., 2004; Sabharwal et al., 2004; McDowall and Dampney, 2006; do Carmo et al., 2007; Kanbar et al., 2007; Kawada et al., 2019
Equation 2	Mthombeni et al., 2012
Equation 3	Mthombeni et al., 2012
Equation 4	Leitch et al., 1997; B = 0: Devanne et al., 1997; Stewart et al., 2021
Equation 5	Cardoso et al., 2005

Ishikawa et al. introduced a fifth parameter (Equation 6) to account for data asymmetry (Ishikawa et al., 1984). According to Ricketts and Head (Ricketts and Head, 1999) Sigmaplot (formerly SPSS now Systat Software Inc.) offers a very similar asymmetric sigmoidal curve fitting equation (Equation 7). The latter authors propose their own asymmetric function (not shown).

The upper part of Figure 2 shows the nomenclature related to functions by taking the example of the Boltzmann sigmoidal function which has 4 parameters (2^*nd*^ column). The lower part of the figure shows example parameter values that have been obtained by parameter optimization (nonlinear curve fitting) to fit the example data as given in Results section “Curve fitting by means of the modified Boltzmann sigmoidal equation to experimental data.” What all these 5 traditional equations have in common is that their Slope parameters (2^*nd*^ column) do *not* represent the slopes of the resultant graphs at their steepest portion at Midrange (see superposed curves in the 3^*rd*^ column) as naïve users might expect.

The misnomer has several drawbacks. First, there is the risk of using the fitting result ‘as is’ by users who are not aware of the mismatch. Second, users who know about the problem that the parameter represents a surrogate only, called, e.g., “gain coefficient” (do Carmo et al., 2007), “coefficient for the determination of gain” (Scrogin et al., 1994), “slope factor” (Shade et al., 1990), “slope coefficient” (Leitch et al., 1997; Kanbar et al., 2007), “slope parameter” (Devanne et al., 1997), “curvature parameter” (Schenberg et al., 1995), “curvature coefficient” (Cardoso et al., 2005), or “coefficient of curvature” (Leitch et al., 1997), have to determine the true value by further calculation. Third, curve fitting algorithms may need decent starting values or intervals for parameter optimization to successfully converge. Suitable presets for Bottom, Top or Range, and Midrange can be visually derived by the user from X-Y plots of the experimental data. Yet, the Slope parameter as visually estimated from the plots deviates from a useful preset. The latter needs further calculation which complicates the preparation step for curve fitting. Forth, scientific novices cannot readily understand why, after successful curve fitting, three of the four parameter values are equivalent to the graph’s characteristics but Slope (baroreflex gain) is not.

Here we propose a modified Boltzmann sigmoidal equation that ensures one-to-one correspondence between the parameter names and the mathematical characteristics of the resultant graphs. The proposal will simplify sigmoidal curve fitting and, in particular, avoid misinterpretation and misreporting of baroreflex sensitivity.

## Methods

Equation 8 is our proposal for a modified Boltzmann sigmoidal equation:

Modified Boltzmann sigmoidal function


(8) $$y=f\left(x\right)\;B+\frac{T-B}{1+e^{\frac{4S\left(M-x\right)}{T-B}}}$$

Linear form of the equation for usage in fitting tools


$$Y=B+\left(T-B\right)/\left\{1+exp\left[4^{*}S^{*}\left(M-X\right)/\left(T-B\right)\right]\right\}$$

**FIGURE 3:**
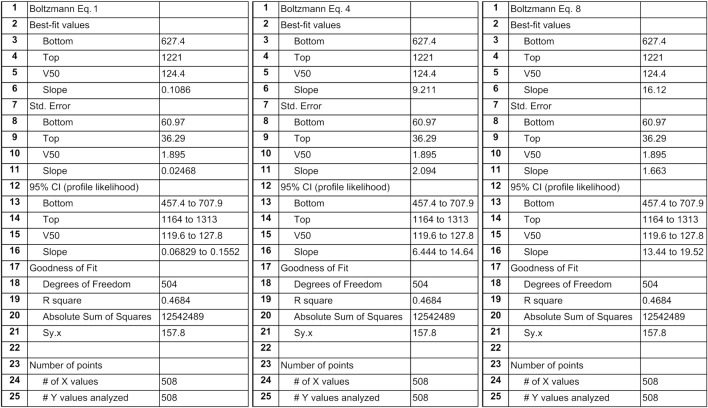
Screenshots of GraphPad^®^ Prism report tables after fitting different Boltzmann sigmoidal functions to real cardiac baroreflex data. We used the nonlinear curve fitting tool of GraphPad Prism (GraphPad, RRID:SCR_002798) to fit the four parameters of the Boltzmann sigmoidal functions related to Equation 1, Equation 4, and Equation 8 to experimentally obtained cardiac baroreflex data from a previous study (Heusser et al., 2016). The result tables report identical values for the optimized parameters Bottom, Top, and Midrange (V50). However, Slopes in line #6 are different. The only Slope that corresponds to the actual slope of the curve (see slope triangle in Figure 4: 16.12 ms/mmHg) is reported by our proposed WYSIWYG Equation 8 as can be expected according to Results section “The [S]lope parameter’s value really represents the slope of the modified Boltzmann sigmoidal curve at the inflection point.” Furthermore, the screenshots exemplify that, after parameter fitting, the resulting graphs are absolutely identical since the Goodness of Fit quantifiers are identical (lines #19–21) which is in agreement with the exactly overlapping curves in Figure 4. Sy.x is a variant of the standard deviation of the residuals that takes the degrees of freedom into account: Sy.x = sqrt [(sum of squared residuals) / (n – degrees of freedom)].

The range T–B can be replaced by R. Then, the four parameters would be B, R, M, and S. The formula is not meant to better fit the data; the goodness of fit is exactly the same as for the other 4-parameter variants of the formula (see superposed curves in Figure 2 and report screenshots in Figure 3). Rather, our intention is to reconcile parameter naming and meaning. In the following, we are going to mathematically prove that the four parameters of the modified equation, namely [B]ottom, [T]op, [M]idrange, and [S]lope, exactly reflect the graph’s characteristics. We will do so point by point with the implicit understanding that [S]lope ≠ 0 and [R]ange = [T]op–[B]ottom > 0.0. Normally, the relationship between RR interval and blood pressure has a positive slope. In contrast, the relationships between heart rate or sympathetic nerve activity and blood pressure feature negative slopes.

## Results

Sections “[B]ottom is the lower limit of the function” through “The [S]lope parameter’s value really represents the slope of the modified Boltzmann sigmoidal curve at the inflection point” prove the correspondence between parameter naming and functional meaning. Captions comment the stepwise proof construction. Section “Threshold and saturation” derives calculation of threshold and saturation pressure. Section “Curve fitting by means of the modified Boltzmann sigmoidal equation to experimental data” exemplifies the usefulness of our proposed equation using real baroreflex data from healthy and diseased subjects, and in section “Practicing curve fitting by means of the modified Boltzmann sigmoidal curve” we invite the readers to test the method on simulated data.

### [B]ottom Is the Lower Limit of the Function

With positive slopes the graphs asymptote to [B]ottom toward the left:

(1) *S* > *0*, *T–B* > *0*

a. $z=\frac{4S\left(M-x\right)}{T-B}$, *x* → −∞ ⟹ *z* → +∞
b.
$\lim_{z\to +\infty }B+\frac{T-B}{1+e^{z}}=B+0=B$

c.
$\lim_{x\to -\infty }B+\frac{T-B}{1+e^{\frac{4S\left(M-x\right)}{T-B}}}=B$

d.
$\lim_{x\to -\infty }f\left(x\right)=B$

With negative slopes the graphs asymptote to [B]ottom toward the right:

(2) *S* < *0*, *T–B* > *0*

a. $z=\frac{4S\left(M-x\right)}{T-B}$, *x* → +∞ ⟹ *z* → +∞
b.
$\lim_{z\to +\infty }B+\frac{T-B}{1+e^{z}}=B+0=B$

c.
$\lim_{x\to +\infty }B+\frac{T-B}{1+e^{\frac{4S\left(M-x\right)}{T-B}}}=B$

d.
$\lim_{x\to +\infty }f\left(x\right)=B$

### [T]op Is the Upper Limit of the Function

With positive slopes the graphs asymptote to [T]op toward the right:

(3) *S* > *0*, *T–B* > *0*

a. $z=\frac{4S\left(M-x\right)}{T-B}$, *x* → +∞ ⟹ *z* → −∞
b.
$\lim_{z\to -\infty }B+\frac{T-B}{1+e^{z}}=B+\frac{T-B}{1+0}=B+T-B=T$

c.
$\lim_{x\to +\infty }B+\frac{T-B}{1+e^{\frac{4S\left(M-x\right)}{T-B}}}=T$

d.
$\lim_{x\to +\infty }f\left(x\right)=T$

With negative slopes the graphs asymptote to [T]op toward the left:

(4) *S* < *0*, *T–B* > *0*

a. $z=\frac{4S\left(M-x\right)}{T-B}$, *x* → −∞ ⟹ *z* → −∞
b.
$\lim_{z\to -\infty }B+\frac{T-B}{1+e^{z}}=B+\frac{T-B}{1+0}=B+T-B=T$

c.
$\lim_{x\to -\infty }B+\frac{T-B}{1+e^{\frac{4S\left(M-x\right)}{T-B}}}=T$

d.
$\lim_{x\to -\infty }f\left(x\right)=T$

### [M]idrange (V50) Is the Abscissa of an Inflection Point

Using the abbreviations


[R]ange=[T]op-[B]ottomand

$$\left[G\right]radient=4*\left[S\right]lope\to \left[S\right]lope=\frac{G}{4}$$

our proposed equation can be written as


(9) $$f\left(x\right)=B+\frac{R}{1+e^{\frac{G\left(M-x\right)}{R}}}$$

To prove the assertion we need the first, second, and third derivatives. They are:


(10) $$f^{\prime }\left(x\right)=\frac{R\left(-1\right)\left(-\frac{G}{R}\right)e^{\frac{G\left(M-x\right)}{R}}}{{\left(1+e^{\frac{G\left(M-x\right)}{R}}\right)}^{2}}=\frac{G*e^{\frac{G\left(M-x\right)}{R}}}{{\left(1+e^{\frac{G\left(M-x\right)}{R}}\right)}^{2}}$$

(11) $$f^{\prime \prime }\left(x\right)=\left(\frac{G^{2}}{R}\right)\left(\frac{e^{\frac{G\left(M-x\right)}{R}}\left[-1+e^{\frac{G\left(M-x\right)}{R}}\right]}{{\left[1+e^{\frac{G\left(M-x\right)}{R}}\right]}^{3}}\right)$$

(12) $$f^{\prime \prime \prime }\left(x\right)=\left(\frac{-2G^{3}}{R^{2}}\right)\left(\frac{e^{\frac{G\left(M-x\right)}{R}}\left[1-e^{\frac{G\left(M-x\right)}{R}}+e^{{\left(\frac{G\left(M-x\right)}{R}\right)}^{2}}\right]}{{\left[1+e^{\frac{G\left(M-x\right)}{R}}\right]}^{4}}\right)$$

Below we outline that the necessary but not sufficient condition for [M]idrange to be the abscissa of an inflection point of the modified Boltzmann sigmoidal equation, namely *f*″ (*M*) = 0, is fulfilled. Note that [M]idrange is passed as x argument to Equation 11:


$$f^{\prime \prime }\left(M\right)=\left(\frac{G^{2}}{R}\right)\left(\frac{e^{\frac{G\left(M-M\right)}{R}}\left[-1+e^{\frac{G\left(M-M\right)}{R}}\right]}{{\left[1+e^{\frac{G\left(M-M\right)}{R}}\right]}^{3}}\right)$$

$$\begin{array}{c} f^{\prime \prime }\left(M\right)=\left(\frac{G^{2}}{R}\right)\left(\frac{e^{0}\left[-1+e^{0}\right]}{{\left[1+e^{0}\right]}^{3}}\right)=\left(\frac{G^{2}}{R}\right)\left(\frac{1\left[-1+1\right]}{{\left[1+1\right]}^{3}}\right)\\ \;=\left(\frac{G^{2}}{R}\right)\left(\frac{0}{8}\right)=0 \end{array}$$

Given that we disallow [G]radient, which is 4^∗^[S]lope, to be zero, the sufficient condition for [M]idrange to be the abscissa of an inflection point, namely *f*‴(*M*) ≠ 0, is fulfilled, too, as demonstrated below. Note that [M]idrange is passed as x argument to Equation 12:


$$f^{\prime \prime \prime }\left(M\right)=\left(\frac{-2G^{3}}{R^{2}}\right)\left(\frac{e^{\frac{G\left(M-M\right)}{R}}\left[1-e^{\frac{G\left(M-M\right)}{R}}+e^{{\left(\frac{G\left(M-M\right)}{R}\right)}^{2}}\right]}{{\left[1+e^{\frac{G\left(M-M\right)}{R}}\right]}^{4}}\right)$$

$$\begin{array}{c} f^{\prime \prime \prime }\left(M\right)=\left(\frac{-2G^{3}}{R^{2}}\right)\left(\frac{e^{0}\left[1-e^{0}+e^{0^{2}}\right]}{{\left[1+e^{0}\right]}^{4}}\right)\\ \;=\left(\frac{-2G^{3}}{R^{2}}\right)\left(\frac{1\left[1-1+1\right]}{{\left[1+1\right]}^{4}}\right)=\left(\frac{-2G^{3}}{R^{2}}\right)\left(-\frac{1}{16}\right) \end{array}$$

$$f^{\prime \prime \prime }\left(M\right)\neq 0$$

### The Point at x = [M]idrange (V50) Is the Only Inflection Point of the Function

In order for our proposed function to have only one inflection point, *f*″(*x*) = 0 must be true for only one x value. To show that this is the case, we reuse Equation 11 while highlighting two crucial terms by enclosing them in curly brackets:


$$f^{\prime \prime }\left(x\right)=\left(\frac{G^{2}}{R}\right)\left(\frac{\left\{e^{\frac{G\left(M-x\right)}{R}}\right\}\left\{-1+e^{\frac{G\left(M-x\right)}{R}}\right\}}{{\left[1+e^{\frac{G\left(M-x\right)}{R}}\right]}^{3}}\right)$$

*f*″ (*x*) may become zero if at least one of the factors shown in curly brackets above becomes zero.

As *e^z^ for all z* ∈ ℝ cannot be zero, we have to figure out how to zero the factor on the right:


$$\begin{array}{c} -1+e^{\frac{G\left(M-x\right)}{R}}=0\Leftrightarrow e^{\frac{G\left(M-x\right)}{R}}=1\Rightarrow \frac{G\left(M-x\right)}{R}=0\\ \;\Rightarrow \text{M–x}=0\\ \;x=M \end{array}$$

Calculating the function’s value for x = [M]idrange using Equation 8


$$\begin{array}{c} y=f\left(M\right)=B+\frac{T-B}{1+e^{\frac{4S\left(M-M\right)}{T-B}}}=B+\frac{T-B}{1+e^{0}}\\ \;=B+\frac{T-B}{2}=\frac{B+T}{2} \end{array}$$

shows that it is halfway between the limits [B]ottom and [T]op in analogy to the logistic function prototype whose value is 0.5 at its inflection point (Figure 1).

### The [S]lope Parameter’s Value Really Represents the Slope of the Modified Boltzmann Sigmoidal Curve at the Inflection Point

Here we reuse the abbreviations and first derivative as outlined for Equation 9 in section “[M]idrange (V50) is the abscissa of an inflection point”:


[R]ange=[T]op-[B]ottomand

$$\left[G\right]radient=4*\left[S\right]lope\to \left[S\right]lope=\frac{G}{4}$$

$$f^{\prime }\left(x\right)=\frac{G*e^{\frac{G\left(M-x\right)}{R}}}{{\left(1+e^{\frac{G\left(M-x\right)}{R}}\right)}^{2}}$$

and we pass [M]idrange as x argument to the first derivative:


$$f^{\prime }\left(M\right)=\frac{G*e^{\frac{G\left(M-M\right)}{R}}}{{\left(1+e^{\frac{G\left(M-M\right)}{R}}\right)}^{2}}=\frac{G*e^{0}}{{\left(1+e^{0}\right)}^{2}}=\frac{G}{{\left(1+1\right)}^{2}}=\frac{G}{4}$$

$$f^{\prime }\left(M\right)=S$$

### Threshold and Saturation

**FIGURE 4:**
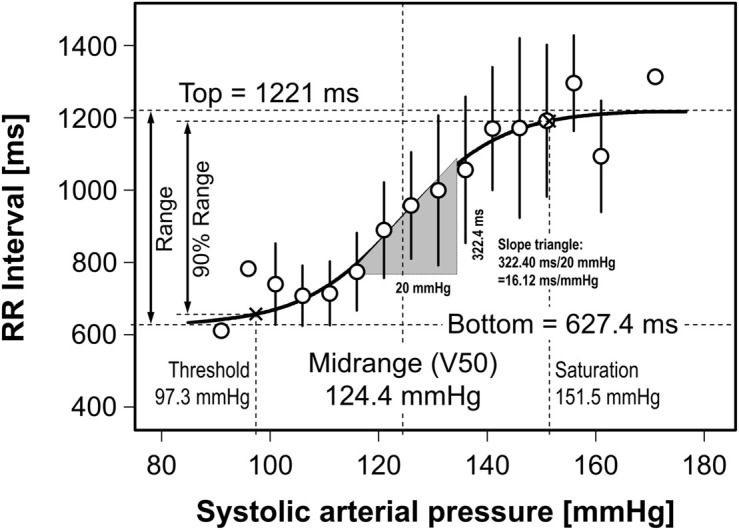
Fitting different Boltzmann sigmoidal functions to real cardiac baroreflex data. Sigmoidal curve fitting to real data from a previous study (Heusser et al., 2016) and illustration of related terminology. After fitting the four parameters of three different equations to these data the resultant function curves overlap exactly. The slope triangle denotes the maximum slopes of the three sigmoidal curves at their inflection point. See Results “Curve fitting by means of the modified Boltzmann sigmoidal equation to experimental data” for more information.

Midrange is an abscissa-related curve characteristic. In our examples (Figures 2, 4), it is the arterial pressure around which pressure disturbances are effectively buffered by the reflex response. Bottom and Top are distinct ordinate values that represent the upper and lower limits of the reflex response. Abscissa values corresponding to Bottom and Top could indicate the pressure range in which the reflex can operate. Unfortunately, such values do not exist for mathematical reasons, since sigmoidal functions show asymptotic behavior against Bottom and Top. The practical solution was to define Threshold and Saturation as the abscissas, where the sigmoidal curve crosses the 5 and 95% margins of the function’s range (Range = Top – Bottom) (Sabharwal et al., 2004; McDowall and Dampney, 2006). Consequently, the ordinate interval related to Threshold and Saturation covers 90% of the reflex response range (Figure 4). The following derivation is guided by a corrective proposal (McDowall and Dampney, 2006).

To calculate Threshold and Saturation the first step is to rearrange our proposed Equation 8 to solve for x:


$$\begin{array}{rcl} y=f\left(x\right)=B+\frac{T-B}{1+e^{\frac{4S\left(M-x\right)}{T-B}}} & ⟹ & y-B=\frac{T-B}{1+e^{\frac{4S\left(M-x\right)}{T-B}}}⟹\\ \frac{T-B}{y-B}=1+e^{\frac{4S\left(M-x\right)}{T-B}} & ⟹ & \frac{T-B}{y-B}-1=e^{\frac{4S\left(M-x\right)}{T-B}}⟹\\ \ln\left(\frac{T-B}{y-B}-1\right)=\frac{4S\left(M-x\right)}{T-B} & ⟹ & \frac{\ln\left(\frac{T-B}{y-B}-1\right)*\left(T-B\right)}{4S}\\ =M-x⟹ \end{array}$$

$$x=M-\frac{\ln\left(\frac{T-B}{y-B}-1\right)*\left(T-B\right)}{4S}$$

In the second step, we have to pass Bottom + 5% of Range as y argument:


$$x=M-\frac{\ln\left(\frac{T-B}{B+0.05\left(T-B\right)-B}-1\right)*\left(T-B\right)}{4S}$$

$$x=M-\frac{\ln\left(\frac{T-B}{0.05\left(T-B\right)}-1\right)*\left(T-B\right)}{4S}$$

$$x=M-\frac{\ln\left(\frac{1}{0.05}-1\right)*\left(T-B\right)}{4S}$$

$$x=M-\frac{\text{ln}\left(19\right)*\left(T-B\right)}{4S}$$

$$x=M-\frac{2.944*\left(T-B\right)}{4S}$$

$$x=M-0.7361*\frac{T-B}{S}$$

In the third step, we have to pass Bottom + 95% of Range as y argument:


$$x=M-\frac{\ln\left(\frac{T-B}{B+0.95\left(T-B\right)-B}-1\right)*\left(T-B\right)}{4S}$$

Intermediate steps as above.


$$x=M+0.7361*\frac{T-B}{S}$$

Result summary: Threshold and Saturation can be calculated using the formula


$$\begin{array}{c} x=Midrange\pm 0.7361*Range/Slope \end{array}$$

Passing the data obtained by curve fitting (Figure 4)


x=124.4mmHg±0.7361*593.6ms/16.12ms/mmHg

x=124.4mmHg±0.7361*36.8mmHg

x=124.4mmHg±27.1mmHg

results in *Threshold pressure = 97.3 mmHg* and *Saturation pressure = 151.5 mmHg*.

### Curve Fitting by Means of the Modified Boltzmann Sigmoidal Equation to Experimental Data

Cardiac baroreflex data have been experimentally obtained earlier in a double-blind, randomized, cross-over study in healthy subjects (Heusser et al., 2016). Stepwise infusions of the vasodilator sodium nitroprusside and the vasoconstrictor phenylephrine elicited blood pressures changes over a large range which is needed for baroreflex *curve* construction. Comparison of these curves after intake of placebo (see Figure 4) and ivabradine (not shown) challenged the so-called use-dependence of ivabradine and might explain its potential for untoward effects. Such an insight would not have been possible with spontaneous methods for baroreflex quantification.

The data points in Figure 4 relate RR intervals and systolic pressures. They have been binned in 5-mmHg intervals. Bin means are represented as open circles and standard deviations as error bars. Circles related to bins with only one data point lack error bars. Curve fitting procedures were weighted according to the number of data points in each bin. As examples, three variants of the Boltzmann sigmoidal equation, namely Equations 1, 4 and 8 – the latter being our proposal – have been fitted to the data. All three resulting baroreflex curves exactly overlap. Consequently, the goodness of fit (see lines #19–21 in the screenshots in Figure 3) is identical for the three equations. The inserted slope triangle in Figure 4 pertains to the maximum slope of the curves at their central inflection points. The lengths of the triangle’s sides have been chosen for purely graphical reasons. Their ratio of 322.4 ms/20 mmHg = 16.12 ms/mmHg denotes the cardiovagal baroreflex sensitivity (baroreflex gain). In contrast, the Slope *parameters* reported after curve fitting using traditional versions of the Boltzmann equation (**Equation 1**: +0.1086 and **Equation 4**: +9.211) do *not* correspond to the curve’s actual slope of +16.12 ms/mmHg (line #6 in the screenshots in Figure 3). In contrast, if the modified Boltzmann equation is used all 4 parameter values reflect the graph’s characteristics properly. Hence, only the modified equation features ‘what you see is what you get’ (WYSIWYG).

**FIGURE 5:**
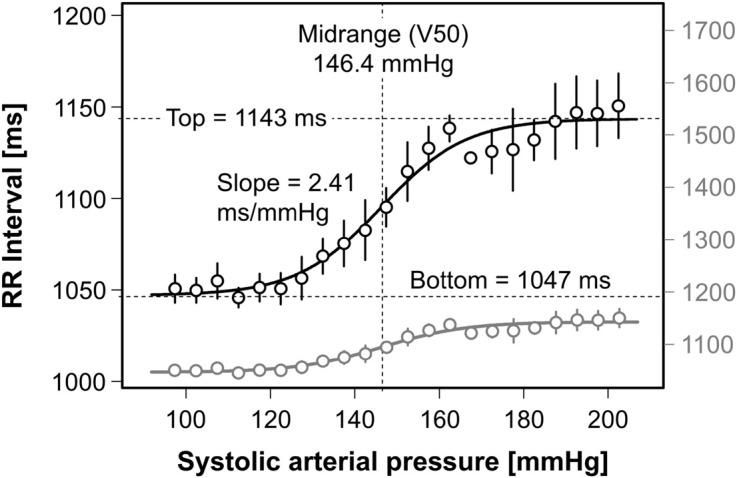
Cardiac baroreflex curve in dysautonomia with marked reductions in response range and baroreflex sensitivity. Sigmoidal curve fitting using Equation 8 has been applied to real data from a patient with dysautonomia. The lower representation of the same data and fitting curve (gray) allows for visual comparison between healthy and diseased subjects because the resolution of the related ordinate on the right (gray) is similar to that in Figure 4. Note the marked reduction in response range (<100 ms) and baroreflex gain (2.41 ms/mmHg).

The modified equation can also be used in patients with disorders of autonomic cardiovascular regulation. The data in Figure 5 represent responses to low-dose injections of sodium nitroprusside (0.25 μg/kg) and phenylephrine (6.25 μg/74 kg) in such a patient. In healthy subjects, these test interventions would hardly change arterial pressure because of the buffering capacity of intact baroreflexes. Yet, in the patient, who also suffers from sympathetic vasoconstrictor incompetence (data not shown), the RR-interval response range is less than 100 ms despite a provoked change in systolic pressure of more than 100 mmHg. Moreover, the baroreflex gain (2.41 ms/mmHg) is much smaller than in healthy subjects. The gray data in Figure 5 are scaled as in Figure 4 for easy visual comparison.

### Practicing Curve Fitting by Means of the Modified Boltzmann Sigmoidal Curve

Interested readers are referred to the Microsoft Excel spreadsheet in the Supplementary Material. The spreadsheet generates two-dimensional data (blood pressure + RR intervals) with adjustable noise. Equation 4 and Equation 8 have been chosen for sigmoidal curve fitting by means of the Excel Solver Add-in. We also suggest to imitate the solver’s attempts to find a good fit by manual adjustments of the equation parameters. By doing so, the reader will realize that adjustments are more direct and easier to achieve when the *modified* Boltzmann equation is used.

## Discussion

Boltzmann sigmoidal equations are frequently used for nonlinear curve fitting to two-dimensional data. Their usefulness has been confirmed for decades. Commonly used forms of the equation have 4 parameters that represent the lower and upper limit or range of the data, the abscissa of the inflection point, and the slope at the latter. They are variants of the formula proposed by Kent et al. (Kent et al., 1972) to provide “a generalized mathematical model of the carotid sinus reflex which contains parameters with meaningful physiological interpretation.” However, while three of the four parameters are directly related to the visible characteristics of the fitting curve, the slope parameter is not. Instead, the actual slope of the curve has to be determined separately. We proposed a modification of the Boltzmann sigmoidal function without this weakness to assist users expecting “what you see is what you get” (WYSIWYG). We mathematically proved that the function’s parameters are one-to-one equivalent to the resultant curve’s characteristics and successfully applied the method to real and artificial baroreflex data. The proposed equation looks slightly more complicated than conventional variants, but it offers some benefits. Once the user has entered the formula in his/her favorite fitting tool, the extra work is loaded onto the computer instead of the user. The traditional mismatch between the slope parameter and the graph’s actual slope is resolved. After the fitting procedure has reached an acceptable result, the reported parameters can be taken as they are computed without any additional postprocessing. The goodness of fit is exactly the same as for the existing variants of the Boltzmann sigmoidal function.

Parameter identifiers like A1..A4 (Kent et al., 1972; Miki et al., 2003; McDowall and Dampney, 2006; do Carmo et al., 2007), P1..P4 (Saad et al., 1989; Itoh and van den Buuse, 1991; Kawada et al., 1992; Bartholomeusz and Widdop, 1995; Sampaio et al., 1999), m1..m4 (Bealer, 2003), a..d (Veelken et al., 1994; He et al., 1999), or a0..a3 (Stewart et al., 2021) are commonly used. More informative names are also assigned to some parameters (Verberne et al., 1987; Schenberg et al., 1995; Cardoso et al., 2005), e.g., BP50, HR_*min*_, and HR_*max*_. The slope parameter, however, continued to be a nomenclatural problem, e.g., “ß is the parameter that governs the slope of the barocurve, i.e., the gain of the baroreflex” (Schenberg et al., 1995) or “dx = a curvature coefficient that is independent of range” (Lee et al., 2002). We hope that our proposal will encourage researchers to embrace meaningful parameter names for sigmoidal curve fitting, including Slope without uncertainty. In our opinion, the more direct identifiers [B]ottom and [T]op as used in Equation 8 should be preferred over [B] and [R]ange, even if we used [R]ange in the Results Section for brevity. In so doing, parameter presetting during the curve fitting preparation step, as often required by fitting tools, is simplified.

Our proposal has the same limitations as the conventional equations. For instance, data asymmetry is not considered (Ishikawa et al., 1984; Ricketts and Head, 1999), and the approach is not able to cope with baroreflex hysteresis (Studinger et al., 2007). The method can only be successfully applied if the blood pressure excursions are large enough to cover the nonlinear parts of the response. Moreover, there is an issue that may occur in vasoactor infusion protocols, namely that the operating pressure *after* stepwise increasing infusion of sodium nitroprusside may be lower than *before*. Thus, subsequent infusion of phenylephrine starts from lower pressures than with nitroprusside infusion. This ‘fracture’ in the baroreflex data needs to be handled before curve fitting to prevent slope overestimation. We used Microsoft Excel and the Excel Solver Add-in to illustrate the ideas behind this work using simulated data (see Supplementary Material) because this spreadsheet software is widely known (Microsoft Excel, RRID:SCR_016137). We do not claim particular suitability or superiority over other tools and did not compare curve fitting capabilities of different tools.

## Conclusion

Using the proposed WYSIWYG variant of the 4-parameter Boltzmann sigmoidal function for nonlinear curve fitting yields exactly the same results as the traditional ones. In contrast, after successful curve fitting, the resultant value for the Slope parameter can be taken “as is” without any further calculation. Thus, usage of the WYSIWYG equation instead of traditional variants is less time-consuming, cumbersome, and error-prone. The equation has a sound mathematical background which promotes correct physiological interpretation of the results. We encourage the reader to benefit from these advantages.

## Data Availability Statement

The original contributions presented in the study are included in the article, further inquiries can be directed to the corresponding author.

## Ethics Statement

The studies involving human participants were reviewed and approved by Ethics Committee of Hannover Medical School Study Code CCB-CRC-07-02, Vote #5223NM. The patients/participants provided their written informed consent to participate in this study.

## Author Contributions

KH contrived the method and wrote the manuscript draft. RH contrived the mathematical proofs. JJ and JT supplied physiological and clinical background. VU and AD checked maths. All authors contributed to the article and approved the submitted version.

## Conflict of Interest

The authors declare that the research was conducted in the absence of any commercial or financial relationships that could be construed as a potential conflict of interest.

## Publisher’s Note

All claims expressed in this article are solely those of the authors and do not necessarily represent those of their affiliated organizations, or those of the publisher, the editors and the reviewers. Any product that may be evaluated in this article, or claim that may be made by its manufacturer, is not guaranteed or endorsed by the publisher.
